# Enhancing African low-resource languages: Swahili data for language modelling

**DOI:** 10.1016/j.dib.2020.105951

**Published:** 2020-06-30

**Authors:** Casper S. Shikali, Refuoe Mokhosi

**Affiliations:** a School of information and Software Engineering, University of Electronic Science and Technology of China., Xiyuan Ave, West Hi-Tech Zone, 611731 Chengdu, Sichuan, PR China; b School of Information and Communication Technology, South Eastern Kenya University, 170-90200, Kitui, Kenya; ⁎ Corresponding author at: School of information and Software Engineering, University of Electronic Science and Technology of China., Xiyuan Ave, West Hi-Tech Zone, 611731 Chengdu, Sichuan, PR China.

**Keywords:** Natural language processing, Deep learning, Language modelling, Unannotated data, Word analogy, Syllables, Neural networks

## Abstract

Language modelling using neural networks requires adequate data to guarantee quality word representation which is important for natural language processing (NLP) tasks. However, African languages, Swahili in particular, have been disadvantaged and most of them are classified as low resource languages because of inadequate data for NLP. In this article, we derive and contribute unannotated Swahili dataset, Swahili syllabic alphabet and Swahili word analogy dataset to address the need for language processing resources especially for low resource languages. Therefore, we derive the unannotated Swahili dataset by pre-processing raw Swahili data using a Python script, formulate the syllabic alphabet and develop the Swahili word analogy dataset based on an existing English dataset. We envisage that the datasets will not only support language models but also other NLP downstream tasks such as part-of-speech tagging, machine translation and sentiment analysis.

**Specifications Table**

**Table:** 

**Subject**	Computer Science, Artificial intelligence
**Specific subject area**	Natural Language Processing, Language modelling
**Type of data**	Text (text file -the unannotated Swahili text are sentences). Table (text file - the Swahili syllabic alphabet is a list of syllables). Table (text file - the word analogy dataset consists of a group of four related words).
**How data were acquired**	Data combines publicly available data by Gelas et al. [6], generation of Swahili syllabic alphabet and transformation of the English word analogy dataset by Mikolov et al. [11]. The unannotated Swahili dataset was derived by processing the dataset by Gelas et al. using a Python script. The Swahili syllabic alphabet was generated using the vowels, syllabification rules by Amidu [1] and, the di-graphs and tri-graphs by Masengo [10]. While the Swahili analogy dataset was developed by translating the English dataset using google translate and replacing the groups without the Swahili equivalence.
**Data format**	Raw
**Parameters for data collection**	Following Mikolov et al. [11] partitions of the Penn tree Bank (PTB)English dataset, we created an unannotated Swahili dataset with train, development and test partitions from the Gelas et al. dataset. The sentences are lowercased with appropriate start and end sentence markers. The size of the dataset was put close to the standard PTB to facilitate comparison to the existing state of art models. The Swahili word analogy dataset was based on the standard English word analogy dataset by Mikolov et al. [11].
**Description of data collection**	An unannotated Swahili data consists of sentences from various Swahili online media platforms. Consequently, the data cuts across various fields such as sports, general news, family, politics and religion. The Sentences are lowercased and partitioned as train, valid and test for language modelling using neural networks which require such partitions for training, hyper-parameter tuning and evaluation of models respectively. The Swahili syllabic alphabet is a list comprising of all possible derived combination of vowels, di-graphs and tri-graphs. The Swahili word analogy dataset consists 12,864 questions of various categories such as family, sounds, singular-plural, countries and cities, counties and constituencies in Kenya, verbs in present continuous tense, transformed verbs, verbs in present and past tense.
**Data source location**	The source of the unannotated dataset is [6]. The Swahili word analogy dataset is partially sourced from Mikolov et al. [11].
**Data accessibility**	Unannotated Swahili data Repository name: Zenodo Data identification number: 10.5281/zenodo.3553423 Direct URL to data: https://doi.org/10.5281/zenodo.3553423 Swahili syllabic alphabet Repository name: Zenodo Data identification number: 10.5281/zenodo.3544180 Direct URL to data: https://doi.org/10.5281/zenodo.3544180 Swahili word analogy dataset Repository name: Zenodo Data identification number: 10.5281/zenodo.3529878 Direct URL to data: https://doi.org/10.5281/zenodo.3529878
**Related research article**	Shikali C. S, Sijie Z, Qihe L, Mokhosi R, 2019 Better word representation vectors using syllabic alphabet: A case study of Swahili. Applied Sciences 9, 3648. doi:10.3390/app9183648

**Value of the Data**

• These datasets are important because they contribute to the available resources for language modelling especially Swahili being a low resource language. In addition, the Swahili word analogy dataset is the only one available for evaluating Swahili language models while the syllabic alphabet will serve as sub-word units with syntactic and semantic meaning hence improve learning in neural networks.
• The datasets will benefit researchers in deep learning and machine learning who specialise in natural language processing. In addition, application developers interested in translation engines, text editors and automatic systems targeting East Africa community will find these datasets very useful.
• Unannotated Swahili dataset can be used for modelling Swahili word representation in NLP tasks based on the words or sub-words. The train partition provides data for optimizing the language models with an aim of minimizing the objective function. The valid partition facilitates adjustment of hyper-parameters to achieve optimal results while the test partition is for evaluating the model. The syllabic alphabet can be used as composition units in language models to derive quality word embeddings that efficiently address the agglutinative nature of Swahili [13]. In addition, the syllables can be used in other NLP tasks such part-of-speech tagging, text classification, parsing, machine translation, sentiment analysis and morphological analysis. On the other hand, the Swahili analogy dataset can be used in a script to evaluate language models to establish the quality of word representation. The average accuracy over the entire corpus provides a measure of word representation with high values indicating good word associations in terms of syntax and semantic.
• The translation engines that could be generated using the dataset will go a long way to enhance the East Africa integration that has been slippery.

## 1 Language modelling

Natural language processing (NLP) involves using computational techniques to learn, understand and produce human language content [7]. In this regard, there are various NLP tasks such as machine translation, sentiment analysis, parsing, text classification, dialogue systems, speech recognition and question/answer systems which rely on language modelling for good performance. Consequently, we have automated analysis of language structure that has resulted into tools for real-world applications, translation engines and systems for emotions towards products and services. Language modelling involves representing words in automated system with the aim of capturing the human language components such as syntax, context, phonemes, morphology and semantics.

Machine learning and deep learning algorithms have been instrumental in NLP [15] with word embeddings playing an important role in the success of language automation systems. These algorithms widely depend on the availability of data. As observed by Hirschberg and Manning [7], NLP resources and systems have been a major limitation for NLP today hence the use of terms such as high resource languages and low resource languages. They further point out that many low resource languages such as Swahili, Bengali and Punjabi are spoken and written by millions but there are inadequate resources or systems available and that the challenge for the language community is how to develop resources and tools [3,4]. We therefore, in this article, contribute resources that will support Swahili language modelling, as well as lay a framework for other NLP downstream tasks.

Swahili is a Bantu language spoken by more than 100 million people in East and Central Africa [3]. Despite the popularity of the language with a lot of speech and text data, Swahili is still classified under low resource language with limited pre-processed open access data [2,3,4]. For this reason, NLP research on Swahili has been limited to the restricted annotated Helsinki dataset [8] that was developed by researchers from Helsinki university in conjunction with university of Nairobi. Although Gelas et al. [6] have availed an unannotated Swahili dataset, this data needs further pre-processing and adjustments to facilitate good comparative analysis against the existing state-of-art NLP models particularly in language modelling. In this article, we leverage on the Gelas et al. [6] work to provide pre-processed, unannotated Swahili dataset that has standard splits for training, validation and testing. Further, we avail a Swahili syllabic alphabet with a finite vocabulary that could be used for building Swahili word embeddings when applying compositional units to address the agglutination feature of Swahili [13].

Good word representation in language models must be analysed for verification. According to Mikolov et al. [11], one of the quantitative analytical techniques is using word analogy task where the relationships among the words is assessed. This test involves a triplet of words with the goal of guessing the fourth word. That is, given the representation vectors of words A, B and C then the vector of D can be derived by X_B_ - X_A_ + X_C_ where X_i_ represents the word representation vector of word i. However, this test cannot be applied on Swahili language models because of non-existence of a Swahili analogy dataset. We therefore present a Swahili analogy dataset that was developed based on the English dataset introduced by Mikolov et al. [11].

In this article, we provide three datasets that are meant for language modelling using neural networks. The datasets are:

• Unannotated Swahili dataset - these are lowercase sentences with sentence markers.
• Swahili Syllabic alphabet - a list of syllables that form the basis of Swahili words.
• Swahili word analogy dataset - a group of four related words.

The details of the datasets are provided in the data description section.

## 2 Data description

This section provides an individual description of each dataset in the following subsections.

### 2.1 Unannotated Swahili dataset

Table 1 shows the partitions and their corresponding sizes of the Swahili dataset developed specifically for language modelling task. The dataset contains 28,000 unique words with 6.84 M, 970k, and 2 M words for the train, valid and test partitions respectively which represent the ratio 80:10:10. The entire dataset is lowercased, has no punctuation marks and, the start and end of sentence markers have been incorporated to facilitate easy tokenization during language modelling. The train partition is the largest in order to support unsupervised learning of word representations while the hyper-parameters are adjusted based on the performance on the valid partition before evaluating the language model on the test partition. We supply the dataset on the link https://doi.org/10.5281/zenodo.3553423 that contains the train, valid and test partitions.

**Table 1:** The details of unannotated Swahili datasets derived from Gelas et al.[6]. The derived dataset, our dataset, consists of lowercased sentences partitioned into train, valid and test. The sentences have start and end of sentence markers.

Dataset	Unique words	Train	Valid	Test
Gelas et al.	512k	204M	1.8M	1.09M
Derived	28k	6.84M	970k	2M

### 2.2 Swahili syllabic alphabet

We present the entire syllabic alphabet as used in Shikali et al. [13] whose syllables are the foundation of Swahili morphemes and words. The syllables include prefixes that serve as Swahili noun class markers or subject prefixes (*a, wa, vi, ki, m, mi*), tense prefixes (*na, li, ta, nge*), relative prefixes (*o, mo, ko, po, cho, vyo, lo, ye*) and object markers (*ki, vi, m, wa, mwu, ya, ji, mu, kwa, zi*). Therefore, the syllables carry syntactic and semantic meanings which are important in natural language processing. The alphabet explicitly captures the digraphs, tri-graphs and special syllables as observed by Masengo [10] and Polome [12]. The composed Swahili syllables are listed from the four-letter syllables to single-letter (special) syllables. We supply the syllables on the link https://doi.org/10.5281/zenodo.3544180.

### 2.3 Swahili word analogy dataset

Word analogy test, used to evaluate a language model, was introduced by Mikolov et al. [11] to check the quality of word embeddings generated by a neural network language model. Therefore, we leverage on their English word analogy dataset to present a Swahili analogy dataset that consists of 12,864 questions. The dataset is organized into 12 categories whose details are populated in Table 2. We supply the dataset on the link https://doi.org/10.5281/zenodo.3529878.

**Table 2:** Description of the categories of Swahili word analogy dataset.

Category	Description
Jiji-Nchi	These are names of capital cities with their corresponding countries.
Mji-Jimbo	Names of towns and corresponding county in Kenya.
Eneo- Jimbo	Outlines constituencies with their corresponding county in Kenya.
Tanakali	These are sounds produced by various actions.
Familia	Names associated to various family members by gender.
Mnyambuliko wa vitenzi	Transformations of Swahili verbs.
Ukanushaji	Negation of various Swahili verbs.
Mzizi-Kitenzi	Swahili stems with their corresponding verbs.
Nchi-Hulgha	Outlines names of countries with their corresponding general culture.
Wakati uliopo- iliopita	These are present tense verbs with their corresponding past tense.
Umoja - wingi	Consists of singular and plural forms of various nouns.
Wakati endelezi	Simple tense verbs with their corresponding present continuous tense.

## 3 Experimental design, materials and methods

In this section, we describe the methodology used to prepare the datasets with an aim of facilitating easy data assimilation or usage. Therefore, we describe the procedure for developing the pre-processed Swahili unannotated dataset, syllabic alphabet and Swahili word analogy dataset in the following sub-sections.

### 3.1 Processing the unannotated Swahili dataset

We used the work of Gelas et al. [6] to prepare an unannotated Swahili corpus that would provide an event space which is comparable with other existing corpora for language modelling [9], [14]. Gelas et al. had collected Swahili sentences from Tanzania-based online newspapers; the data was made up of 28 million words with a wide scope that included sports, general news, religion and family life. Therefore, we obtained our dataset by pre-processing the train partition of the [6] corpus using a Python script (*preprocess.py*) which lowercased, removed punctuation marks and incorporated start and end of sentence markers, as well as replaced singletons with <unk>; the pseudocode of the script is found in Table 3. Following Mikolov et al. [11], we used a conditional statement in the Python script to reduce rare words where any word with a count of less than 2 was replaced with 〈UNK〉 token. Further, we partitioned the resulting dataset in the ratio 80:10:10 as train, valid and test data respectively following De Pauw et al. [5]. It should be noted that tokenization of the datasets was not done to allow various input strings depending on the model requirements. Table 1 outlines the details of the dataset.

**Table 3:** The pseudocode for the preprocess.py Python script used to pre-process the dataset by Gelas et al. [6].

Open the Gelas' training dataset for reading
Open train, development and test for writing
Read gelas' training file
Lowercase, remove punctuation marks and add sentence markers
if wordcount < 2 then replace with "unk"
Partition the processed file in the ratio 80:10:10
Write to train, development and test file respectively

### 3.2 Processing the Swahili syllabic alphabet

The syllabic alphabet outlines all the possible combination of consonants and vowels that serve as a basis for all the Swahili words. According to Polome [12], Swahili uses *a, e, i, o* and *u* as vowels and all the English alphabet except *x* and *q* as consonants. The syllables are considered the smallest unit in Swahili [12] and could consists of a vowel preceded by one to three consonants though there are special syllables made of single consonants or vowels. To derive the Swahili syllabic alphabet, we used the syllabification rules by Amidu [1] and the following digraphs and tri-graphs by Masengo [10].

*a, b, bw, ch, d, dh, e, f, g, gw, h, i, j, jw, k, kh, l, lw, m, mw, n, ng, ng', nj, nw, ny, o, p, pw, r, s, sw, sh, t, tw, th, u, v, vy, w, y, z, zw, ndw, chw, nyw, ngw, mbw, njw, nzw, shw*

### 3.3 Processing the Swahili word analogy dataset

We generate a Swahili word analogy dataset based on the English dataset developed by Mikolov et al. [11]. Therefore, we translated a few of the categories in the English dataset to Swahili except for comparatives, superlatives, antonyms and city-in-state which were replaced with peculiar Swahili categories. Some of the peculiar categories that were added include sounds (*tanakali*), names of counties in Kenya with their corresponding towns and constituencies, Verb transformations and singular-plural forms. The dataset was then reviewed by two people to confirm the translations and the various new categories that were introduced.

## Declaration of Competing Interest

The authors declare that they have no known competing financial interests or personal relationships which have, or could be perceived to have, influenced the work reported in this article.

## Appendix Supplementary materials

Image, application 1

Image, application 2
