# Supplementary material for: Enhancing African low-resource languages: Swahili data for language modelling
Source: Data Brief. 2020 Jun 30;31:105951. doi: 10.1016/j.dib.2020.105951 (PMC7339006; doi:10.1016/j.dib.2020.105951)
Supplement: Supplementary file 2 [file mmc2.docx]

1. Casper S. Shikali - Conceptualization of this study, Methodology, Programming, Typesetting
2. Refuoe Mokhosi - Programming
